# Medical waste generation and management in medical clinics in South of Iran

**DOI:** 10.1016/j.mex.2019.03.029

**Published:** 2019-04-03

**Authors:** Mohammad Hadi Dehghani, Hamid Dashti Ahrami, Ramin Nabizadeh, Zoha Heidarinejad, Ahmad Zarei

**Affiliations:** a Department of Environmental Health Engineering, School of Public Health, Tehran University of Medical Sciences, Tehran, Iran; b Institute for Environmental Research, Center for Solid Waste Research, Tehran University of Medical Sciences, Tehran, Iran; c Center for Air Quality Research, Institute for Environmental Research (IER), Tehran University of Medical Sciences, Tehran, Iran; d Department of Environmental Health Engineering, Faculty of Health, Hormozgan University of Medical Sciences, Bandar Abbas, Iran; e Food Health Research Center, Hormozgan University of Medical Sciences, Bandar Abbas, Iran; f Department of Environmental Health Engineering, Faculty of Health, Social Development and Health Promotion Research Center, Gonabad University of Medical Sciences, Gonabad, Iran

**Keywords:** Quantitative and qualitative analysis of medical wastes, Waste management, Medical clinics, South of Iran

## Abstract

Medical wastes account for around 1–2% of urban wastes, which are very important in terms of health. In this regard, they are very important and can jeopardize human health. The aim of this study was to determine the qualitative and quantitative characteristics of the wastes in medical clinics in the south of Iran and in order to present suitable management solutions. First, 14 medical clinics were chosen and 24 samples were taken from each clinic (two samples per month) with a total 336 samples. Considering the special properties and the risk potential, the wastes generated in medical clinics were categorized as infections and special waste groups. In terms of properties, they were classified as pseudo-household, infectious, sharp, pharmaceutical, and paper wastes. Once the samples were collected, they were weighed and the results were analyzed by SPSS. The results indicated that in terms of quantity, the waste generated in the first and second groups was 8550.377 and 8053.71 kg/year, respectively. Furthermore, most of the wastes generated in the first and second groups accounted for pseudo-household (80.7%) and infectious (72.77%) wastes, respectively. Due to presence of the specialty of pathology laboratory in the second group, the quantity of infectious waste has increased. Therefore, for proper management of medical wastes in the studied clinics, the clinics of the studied study should implement and apply the rules of waste management properly. Furthermore, training physicians and employees in clinics about reducing, recycling, and collecting wastes in a separate form in clinics should be done in priority.

• In this study, the classification of Basel convention and World Health Organization was considered as the basis of waste classification.
• The results indicated that in the first group of the studied clinics, the order of the waste quantity was as follows: pseudo-household > infectious > sharp > paper.
• Due to large amounts of hazardous infectious wastes in the second group of the studied medical clinics, it necessitates proper management of collection and disposal of these wastes.
• Results can be used to improve the management of waste generation practices in medical clinics with high risk and special wastes potential.

## Graphical abstract

**Figure:**
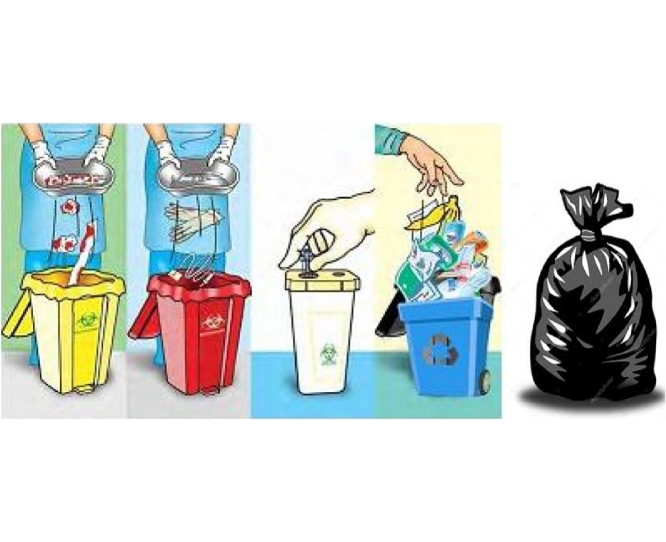

**Specifications Table**

**Table:** 

Subject area:	Environmental science; Medical clinics wastes
More specific subject area:	Medical waste management
Protocol name:	quantitative and qualitative analysis of medical wastes
Name and reference of original method:	The codes and instructions approved and announced in Iran were also considered. Furthermore, the sampling method was chosen based on other studies and the guidelines of American Environmental Protection Agency.
Resource availability:	data

## Description of protocol

The rapid population growth and industrialization has increased the quantity of waste generation [[1], [2], [3], [4]]. Among wastes, medical wastes has become a critical issue as they induce potential health problems and damage to the environment [[5], [6]].

## Details of the method

The present study is a descriptive cross-sectional research conducted with the aim of determining the characteristics and quantity of wastes in medical clinics in the south of Iran. In this study, the classification of Basel convention and World Health Organization was considered as the basis of waste classification [[6], [8]]. In addition to the criteria presented in these instructions, the rules, regulations, codes, and instructions approved and announced in Iran were also taken into account [4]. The sampling method was also selected based on previous studies as well as the guidelines of the United states Environmental Protection Agency [11]. In this study, random sampling was performed in the clinics. In the sampling was conducted two times monthly. The samples were taken in the weeks with the minimum non-holiday working days. 14 medical clinics were chosen and 24 samples were taken from each clinic (two samples per month). Since the selected sample size includes 14 clinics, totally 336 samples were taken from the clinics. In this study, due to ethical considerations, the name of the studied city has not been mentioned.

## The research context and classification of clinics

In the studied region, since provision of healthcare and special services is performed mostly in the capital of the province, thus the patients in different parts of the province were referred to the capital of the province to benefit from these services. Accordingly, the clinics in this study were very crowded. The medical waste generation centers in the studied region, which were sampled and analyzed were classified into the two following groups and investigated further based on the risk potential and the quantity of the generated wastes as:

A: the first group included clinics which generate infectious wastes. These clinics include orthopedic, Ear, Nose and Throat (ENT), gynecology, urology, dermatology, ophthalmology, and surgery specialties.

B: second group: it includes clinics which generate special wastes. These clinics include radiology and pathology specialties along with medical diagnostic laboratories.

## Wastes collection and separation

First, the wastes generated in the clinics were classified according to their characteristic as sharp, infectious, pharmaceutical, pseudo-household, and paper. Different components of waste were separated according to Table 1. When visiting the clinic, they were requested to separate their wastes in the point of generation according to the instructions. This means that they should use five different containers for separating their wastes. The special containers for waste separation in were provided as follows:

1 A yellow plastic bag, for infectious wastes
2 Safety box, for sharp wastes
3 Blue plastic bag, for pharmaceutical wastes
4 Black plastic bag, for pseudo-household wastes
5 White plastic bag, for paper

**Table 1:** Classification of different components of the waste of medical clinics studied.

Group	Components
Pseudo-household wastes	Tissue paper, dry gas and cotton, paper with nylon coating, coating of syringe and needle package, cardboard, fabrics, disposable cups, paper tape, glass adhesive tape, remnants of foods and beverages, orthopedic plaster, tea waste, laboratory kit covers, plastic, nylon, cigarette filter, food packaging, fruits skins
Infectious wastes	Blood, mucus, feces, urine of patients, clot tube, different small, medium and large disposable test tubes, culture medium of stool, urine, mucus, the specimen container of stool, urine, mucus, and pap smear slides, vials, diagnostic kits, oxalate container, CBC, tissue paper, cotton and gas contaminated with blood and other patient secretions, nylon gloves, latex gloves, suture, plastic syringe, tongue depressors, pipette tips, applicator, clot tube
Sharp	Syringe and hypodermic needle, surgical blades, suture needles, lancet, scalpel, broken vials, slides and microscopic slides, broken test tubes
Pharmaceutical	Expired drugs, drug residues, broken thermometers, film processing drugs
Paper	Paper, newspaper, the paper of insurance booklet, the paper of lab results, the visit paper

## Analytical method

At the end of the working day, the researcher referred to each clinic to take the samples. A special label was attached on each of the waste collection containers, and specifications including the name and address of the producer, type of waste, date of generation and collection were written. Then, the samples were transferred to a suitable place by a vehicle for performing physical analysis and then immediately analyzed. Since the wastes contain the blood and stool samples of patients as well as other secretions, they are highly absorbent of insects and the physical analysis (weighing by a scale) should be performed in a suitable place in order to prevent disturbance caused by insects. During the analysis, suitable work clothes, mask, tarpaulin gloves, goggles, and suitable forceps were used. Each group of the wastes inside their special container which had been separated in the clinic was weighed separately and the number obtained from the weighing was recorded in a special form. The number which is obtained from weighing the separated wastes represents the level of generation of different types of waste at the end of the working day in each clinic.

When taking samples from the clinics, the number of patients referring to each clinic was recorded in a special form in order to obtain the per capita waste generation of each patient based on the total level of waste generation. Through dividing the level of daily waste reduction in each clinic by the number of patients visiting the clinic on that day, the per capita waste generation of each patient on that working day was achieved.

For statistical description, the data obtained in these tables were introduced in SPSS software. As mentioned previously, six samples were taken from different types of wastes generated in each clinic. By taking average from the data obtained from the six times of sampling, the mean daily generation of different types of waste in each clinic was determined (Table 2). In order to specify the level of annual generation of different types of waste in the medical clinics available in the sample size, the mean daily generation of different types of waste was multiplied by the number of working days of clinics which is 288 days to obtain the annual generation level of different types of ways in medical clinics. In order for the results obtained for the sample to be generalizable to the total the statistical population, the generalization coefficients should be calculated. Since medical clinics include different specialties and different number throughout the entire statistical population, and in every medical specialty the amount and properties of the generated wastes are different, thus in each specialty via dividing the total number of the medical units of that specialty in the entire statistical population by the number of clinics of that specialty in the sample size, the generalization coefficient was calculated separately for each specialty.

**Table 2:** The mean and standard deviation of the total wastes generated in the studied medical clinics by individual specialties (g/day).

Group Name	Type of specialty	Number	Mean	Standard deviation	Maximum	Minimum
First group	Orthopedic, ENT, gynecology, urology, surgery, dermatology, and ophthalmology	7	483.89	229.73	1196	116
Second group	Radiology and pathology specialties along with medical diagnostic laboratory	7	3758.26	4001.94	13471	243

Via dividing the obtained coefficient by the annual level of different types of waste in each specialty, the total level of annual generation of different types of waste in that specialty throughout the entire statistical population was obtained. Next, by summing up the annual generation level of all types of waste across all specialties in the statistical population, the total annual level of waste generation across all of the medical clinics was calculated.

Table 3, Table 4, Table 5, Table 6 show the mean and standard deviation of the generated wastes of the studied medical clinics by each individual type of specialty in terms of g/day. The level of generation of different types of waste in the medical clinics in the two studied groups is shown in Table 7, Table 8.

**Table 3:** The mean and standard deviation of the pseudo-household wastes generated by the studied medical clinics by individual specialties (g/day).

Group Name	Type of specialty	Number	Mean	Standard deviation	Maximum	Minimum
First group	Orthopedic, ENT, gynecology, urology, surgery, dermatology, and ophthalmology	7	240.89	133.76	623	42
Second group	Radiology and pathology specialties along with medical diagnostic laboratory	7	571.78	269.71	1462	170

**Table 4:** Estimating the mean and standard deviation of the potentially infectious wastes generated by the studied medical clinics by individual specialties (g/day).

Group Name	Type of specialty	Number	Mean	Standard deviation	Maximum	Minimum
First group	Orthopedic, ENT, gynecology, urology, surgery, dermatology, and ophthalmology	7	155.06	104.132	417	26
Second group	Radiology and pathology specialties along with medical diagnostic laboratory	7	2907.59	3516.3	11380	0

**Table 5:** Estimating the mean and standard deviation of the sharp wastes generated by the studied medical clinics by individual specialties (g/day).

Group Name	Type of specialty	Number	Mean	Standard deviation	Maximum	Minimum
First group	Orthopedic, ENT, gynecology, urology, surgery, dermatology, and ophthalmology	7	73.22	69.23	261	0
Second group	Radiology and pathology specialties along with medical diagnostic laboratory	7	151.11	138.5	467	0

**Table 6:** Estimating the mean and standard deviation of the paper wastes generated by the studied medical clinics by individual specialties (g/day).

Group Name	Type of specialty	Number	Mean	Standard deviation	Maximum	Minimum
First group	Orthopedic, ENT, gynecology, urology, surgery, dermatology, and ophthalmology	7	18.04	20.06	84	0
Second group	Radiology and pathology specialties along with medical diagnostic laboratory	7	127.76	155.53	583	0

**Table 7:** The level of different types of waste generated in the medical clinics of the first studied group (kg/year).

Clinic	Type of waste	Weight (kg/year)	Percent
Orthopedic, ENT, gynecology, urology, surgery, dermatology, and ophthalmology	Pseudo-household	14159.404	80.7
	Potentially infectious	2702.85	15.4
	Sharp	547.32	3.11
	Paper	135.601	0.77
	Total	17545.17	100

**Table 8:** The level of different types of waste generated in the medical clinics of the second studied group (kg/year).

Clinic	Type of waste	Weight (kg/year)	Percent
Radiology and pathology specialties along with medical diagnostic laboratory	Pseudo-household	1606.117	19.94
	Potentially infectious	5861.71	72.77
	Sharp	304.51	3.78
	Paper	281.373	3.49
	Total	8053.71	100

## Solutions for the management of medical wastes in the studied clinics

For the management of the wastes of medical clinics, first the education of physicians and other employees of clinics should be done. Then, the plans for reducing waste generation, separation, recycling, and reusing should be implemented. The plan for reducing waste generation can be fulfilled through using materials and products with a lower risk potential or smaller packaging. Instead of injecting drugs, one can use similar oral drugs. The most important measure which can be adopted for optimal management of medical wastes is that different types of waste can be separated at the point generation. The components and compounds of medical wastes have different properties, where the method of management of each type of waste should be based on these properties. Collection and disposal of medical wastes in a mixed way altogether is not a reliable and proper method. Collection of medical wastes along with other urban wastes is also risky both for citizens’ health and makes processing and recycling of materials difficult.

Out of the various medical waste treatment methods, incineration is the most preferable and common management method for medical waste in Iran and many countries [[12], [13], [14]]. Because sometimes these wastes may not properly separated in hospitals due to mistake of personnel. Incineration is in the rank three in the waste management hierarchy proposed by the USEPA, accompanied by source minimization, reuse, recycling, and disposal/landfilling [15]. But medical waste incinerators usually emit high amounts of poisonous gases such as dioxins and furans which are harmful to human health which must be adequately controlled by using air pollution control facilities to reduce the related health problems and also complaints from nearby inhabitants [16].

For different groups of medical wastes, the following solutions are proposed:

## Pseudo-household wastes

These wastes should be well separated from other wastes and transferred and disposed along with other household wastes through public system on a daily basis by the municipality organization related to the urban waste disposal. According to the Iranian waste law, the municipality must carry and dispose these wastes. Trace amounts of harmless pharmaceutical wastes can also be discharged along with these wastes [17].

## Sharp wastes

Regardless of whether these wastes are contaminated or not, they should be collected in an impenetrable safety box [18,19]. The containers for collecting these wastes should have a sound lid and the materials used for the manufacture of these containers should be rigid and impermeable that in addition to keeping sharp objects they should keep any remnants of the fluids inside the syringes. The methods for the management of these wastes include; liquid disinfection by chlorine 0.5%, autoclave/shredding, encapsulation, incineration and eventually disposal in protected sharps barrels or pits [8,20,21].

## Infectious wastes

These wastes should be collected and kept inside resistant bags in washable and disinfected tanks with a special sign of infectious wastes. The best method for infectious waste disposal is disinfection at the point of generation including use of autoclave and controlled proper disposal or use of incinerator.

## Chemical and pharmaceutical wastes

According to the Basel convention, many common drugs used in the treatment of patients referring to clinics are not considered as hazardous drugs and they can be treated as household wastes and get disposed of accordingly [22]. This group of wastes is found in trace amounts in medical clinics. In radiology clinics, trace amounts of chemical wastes are generated which are recycled for silver recovery. The waste of radiology clinics involves chemicals in the processing of radiographic film. These solutions contain silver and the chemical wastes generated in radiology clinics which were recycled in the clinics studied in this research. The radioactive wastes of these clinics are also managed under the supervision of Atomic Energy Organization of Iran.

Therefore, the amount of chemical and pharmaceutical wastes in medical clinics was negligible, and many of the commonly used drugs used to treat patients referred to the clinics were not considered as hazardous drugs, and this group of medicines were co-disposed of with household wastes.

## Paper wastes

In medical clinics, these wastes are generated in minor amounts and in terms of characteristics, they are similar to pseudo-household wastes. Papers should be separated at source of generation, in order to reduce its contamination and avoid deterioration of its quality [23].

## Conflict of interest

The authors of this article declare that they have no conflict of interests.
